# Taxonomic diversity and functional potential of microbial communities in oyster calcifying fluid

**DOI:** 10.1128/aem.01094-24

**Published:** 2024-12-12

**Authors:** Andrea Unzueta-Martínez, Peter R. Girguis

**Affiliations:** 1 Department of Organismic and Evolutionary Biology, Harvard University 123976 , Cambridge, Massachusetts, USA; Norwegian University of Life Sciences, Ås, Norway

**Keywords:** oyster microbiome, calcification, calcifying fluid, microbial ecology

## Abstract

Creating and maintaining an appropriate chemical environment is essential for biomineralization, the process by which organisms precipitate minerals to form their shells or skeletons, yet the mechanisms involved in maintaining calcifying fluid chemistry are not fully defined. In particular, the role of microorganisms in facilitating or hindering animal biomineralization is poorly understood. Here, we investigated the taxonomic diversity and functional potential of microbial communities inhabiting oyster calcifying fluid. We used shotgun metagenomics to survey calcifying fluid microbial communities from three different oyster harvesting sites. There was a striking consistency in taxonomic composition across the three collection sites. We also observed archaea and viruses that had not been previously identified in oyster calcifying fluid. Furthermore, we identified microbial energy-conserving metabolisms that could influence the host’s calcification, including genes involved in sulfate reduction and denitrification that are thought to play pivotal roles in inorganic carbon chemistry and calcification in microbial biofilms. These findings provide new insights into the taxonomy and functional capacity of oyster calcifying fluid microbiomes, highlighting their potential contributions to shell biomineralization, and contribute to a deeper understanding of the interplay between microbial ecology and biogeochemistry that could potentially bolster oyster calcification.

**IMPORTANCE:**

Previous research has underscored the influence of microbial metabolisms in carbonate deposition throughout the geological record. Despite the ecological importance of microbes to animals and inorganic carbon transformations, there have been limited studies characterizing the potential role of microbiomes in calcification by animals such as bivalves. Here, we use metagenomics to investigate the taxonomic diversity and functional potential of microbial communities in calcifying fluids from oysters collected at three different locations. We show a diverse microbial community that includes bacteria, archaea, and viruses, and we discuss their functional potential to influence calcifying fluid chemistry via reactions like sulfate reduction and denitrification. We also report the presence of carbonic anhydrase and urease, both of which are critical in microbial biofilm calcification. Our findings have broader implications in understanding what regulates calcifying fluid chemistry and consequentially the resilience of calcifying organisms to 21st century acidifying oceans.

## INTRODUCTION

Biomineralization is the process by which organisms form mineralized structures. This process has evolved among taxa as diverse as bacteria, protists, plants, and animals in both marine and terrestrial environments. Biominerals are often used for protection against predators and can serve multiple, additional roles. For example, calcium carbonate (CaCO_3_) formations can act as both protective armors and as lenses to facilitate image capture (1), as pH-buffering systems (2), or as detoxification agents (3). Carbonate biominerals are synthesized from a chemically complex aqueous solution called calcifying fluid that is maintained in a carefully regulated inter-membrane space (bounded by epithelial cells). Biological processes modify calcifying fluid chemistry by increasing carbonate and calcium ion concentrations while elevating pH, to make CaCO_3_ precipitation thermodynamically favorable. Additionally, biological processes also regulate the concentration and speciation of other inorganic ions, polysaccharides, and proteins, all of which can drive the mineral polymorph of the final biomineral (e.g., calcite, aragonite, and vaterite). The molecular mechanisms underlying the regulation of calcifying fluid chemistry have not been fully defined. Elucidating these mechanisms and hence the formation of CaCO_3_ biominerals is essential to our understanding of the evolution of animals with mineralized structures, their contributions to global ocean calcification and long-term carbon fluxes, and their sensitivity to future environmental changes.

The potential role of microorganisms in the regulation of calcifying fluid chemistry and, consequently, calcification in marine invertebrates has been previously considered in some taxa such as sponges (4), corals (5), and bivalves (6, 7). Most marine animals harbor diverse microbial communities comprised of bacteria, archaea, viruses, fungi, and protozoans that reside on or within the animal host. These complex communities can provide numerous services to their animal hosts (reviewed in reference 8). For example, some bivalves in sulfide-rich environments rely on chemosynthetic bacteria to supply fixed carbon for nutrition (reviewed in references 9, 10). There is burgeoning evidence that calcifying bacteria associated with marine sponges may play a role in spicule formation (4). Among cnidarians such as corals, there is a relationship between calcification rate and sunlight (5), which implicates their photosynthetic microbial partners, directly or indirectly, in calcification. With respect to bivalves, it has been hypothesized that microorganisms in calcifying fluid can be responsible for calcium carbonate structures that form away from the shell-secreting mantle of the host bivalve (6, 7). However, despite interest in understanding the potential role of microorganisms in marine invertebrate calcification, the mechanisms by which microbial partners could assist in the maintenance of calcifying fluid chemistry and calcification—within the host bivalve—have not yet been elucidated.

The metabolisms responsible for microbial calcification in biofilms are well documented. Marine microorganisms are physiologically diverse and can collectively catalyze a number of chemical reactions that affect the local pH, alkalinity, and dissolved inorganic carbon concentration (11). Energy-conserving reactions such as aerobic photosynthesis, anoxygenic photoautotrophy, dissimilatory iron reduction, sulfate reduction, and methanogenesis can increase carbonate ion concentration and calcium carbonate saturation state in the extracellular space, leading to calcium carbonate precipitation and the formation of structures like stromatolites (11). That said, it is unknown whether oyster-associated microorganisms can play a similar role in calcifying fluid by indirectly altering the calcifying fluid environment to facilitate calcification.

As such, we aim to better understand the contribution of oyster microbial partners to calcifying fluid chemistry maintenance and hence shell calcification. To this end, we used Eastern oysters (*Crassostrea virginica*) as a model marine organism to investigate the potential role of associated microorganisms in regulating the chemistry in calcifying fluid. Oysters are found along coastlines worldwide, providing critical ecological services (12), sustenance, and income to coastal communities across the globe. It is a 12 billion USD a year fishery (13) that is threatened (to varying degrees) across all life stages by ocean acidification (14, 15), yet as mentioned, we know very little about the role of calcifying fluid-hosted microorganisms in enhancing or hindering oyster shell calcification. Oysters are known to harbor diverse microbial communities that are specific according to tissue type (16) and vary by population (17). Recently, via 16S rRNA gene surveys, Sakowski et al. (18) demonstrated that microbial communities in *C. virginica* calcifying fluid are different from the microorganisms in their surrounding seawater and persist across time. However, the functional roles of calcifying fluid microbial communities in regulating local chemistry and shell formation have not been extensively considered.

To investigate the community composition and functional potential of calcifying fluid microbiomes, we collected oysters from three different sites and used shotgun metagenomics to examine taxonomy and functional potential. First, we assessed community composition by testing whether microbial community composition and structure varied among oyster collection sites (i.e., the different locations from which the oysters were harvested) and surveying the viral community to determine their potential contribution to shaping the calcifying fluid microbiome. Second, we examined the functional potential of these microbial communities to better understand their possible role in enhancing or hindering oyster calcification. We identified how microbiome activity might influence the carbonate chemistry in calcifying fluid by assessing the presence of genes involved in biochemical reactions that alter the carbonate system, as well as identifying key enzymes in bacterial calcium carbonate precipitation, carbonic anhydrase, and urease.

## RESULTS

We purchased adult Eastern oysters (88- to 100-mm shell length), *C. virginica*, from three different oyster farms in the summer of 2022: Thatch Island Oysters in Barnstable, USA (*n* = 5); Island Creek Oysters in Duxbury, U.S.A (*n* = 5); and Chebooktook Oysters in New Brunswick, Canada (*n* = 5). Approximately 0.5 mL of calcifying fluid was aseptically collected from each oyster and used for DNA extractions and shotgun metagenomic sequencing on the Illumina NovaSeq S4 system. To overcome the overabundance of host DNA relative to microbial DNA, we sequenced deeply and removed host reads bioinformatically (see Materials and Methods for details).

**Fig 1:**
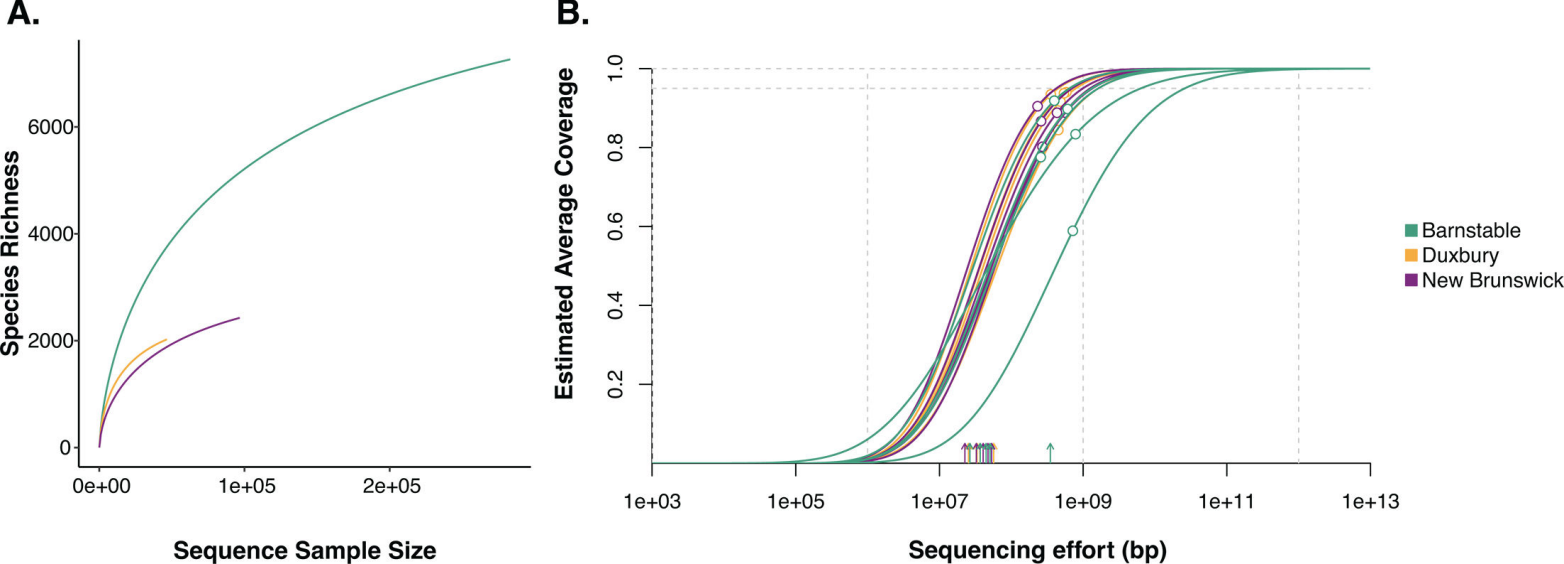
Coverage assessment of oyster calcifying fluid microbiomes. (**A**) Rarefaction curves of the number of species versus the number of sequences in each oyster collection location. Taxonomic annotation was conducted at the contig level. (**B**) Nonpareil curves showing the relationship between estimated coverage and sequencing effort. The white dots on the curves indicate sample estimated coverage. The dashed horizontal lines show the 95% and 100% estimated coverage thresholds, and the vertical arrows along the *x*-axis indicate sample sequencing effort.

After quality filtering and removal of host reads, we retained 53,275,104 read pairs (median, 3,471,027) across all 15 oyster calcifying fluid samples. Metagenomic contigs were assembled and then aligned to the oyster host genome to detect and remove any remaining oyster DNA. A total of 1,197,746 microbial contigs were used for downstream taxonomic and functional potential analyses (see Table S1 in the supplemental material for metagenome metadata). Species discovery rarefaction curves, with taxonomic annotation conducted at the contig level (Fig. 1A), show that species diversity within calcifying fluid microbiomes is not fully captured and indicate that a higher sequencing effort would be needed for complete coverage. However, Nonpareil projection curves (Fig. 1B) estimate coverage values above 75% and 10^7^ – 10^8^ bp sequencing effort for all but one sample; our data recover a large fraction of the sequence diversity allowing characterization of the majority of the taxonomic and functional landscape of calcifying fluid microbiomes. The Nonpareil prediction curves estimate the required sequencing effort at 10–100 Gbp for >95% sequence diversity recovery for calcifying fluid microbiome samples (Fig. 1B).

### Consistency in taxonomic diversity of calcifying fluid microbiomes

**Fig 2:**
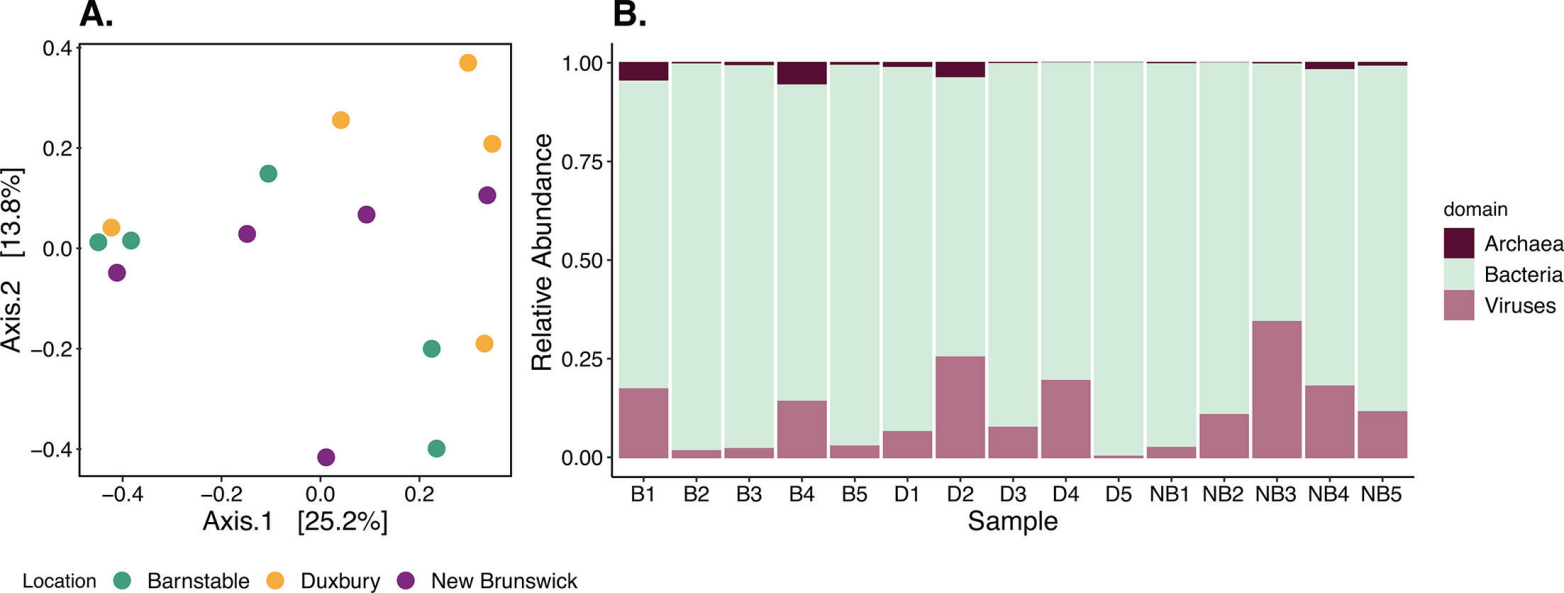
Broad taxonomic overview of archaea, bacteria, and viruses present in oyster calcifying fluid microbiomes. (**A**) PCoA of Bray–Curtis dissimilarity distances (calculated at species level) of microbial communities in calcifying fluid colored by oyster collection location. (**B**) Stacked bar plot of the relative abundance of prokaryotic domains and viruses present in oyster calcifying fluid microbial communities. Relative abundance was calculated at the species level within each sample.

Oyster calcifying fluids harbored microbial communities that were taxonomically highly conserved and not significantly different by oyster collection location (Fig. 2A and B; Table S2). Principal coordinate analysis (PCoA) plots of Bray–Curtis dissimilarities at the species level showed that calcifying fluid microbial communities were equally dispersed across all collection locations (Fig. 2A). Permutational multivariate analysis of variance (PERMANOVA) model confirmed that calcifying fluid microbial community structure was not significantly different according to oyster collection location (Table S2), though there were modest compositional differences among the individual oysters.

**First description of archaea and viruses in oyster calcifying fluid**

**Fig 3:**
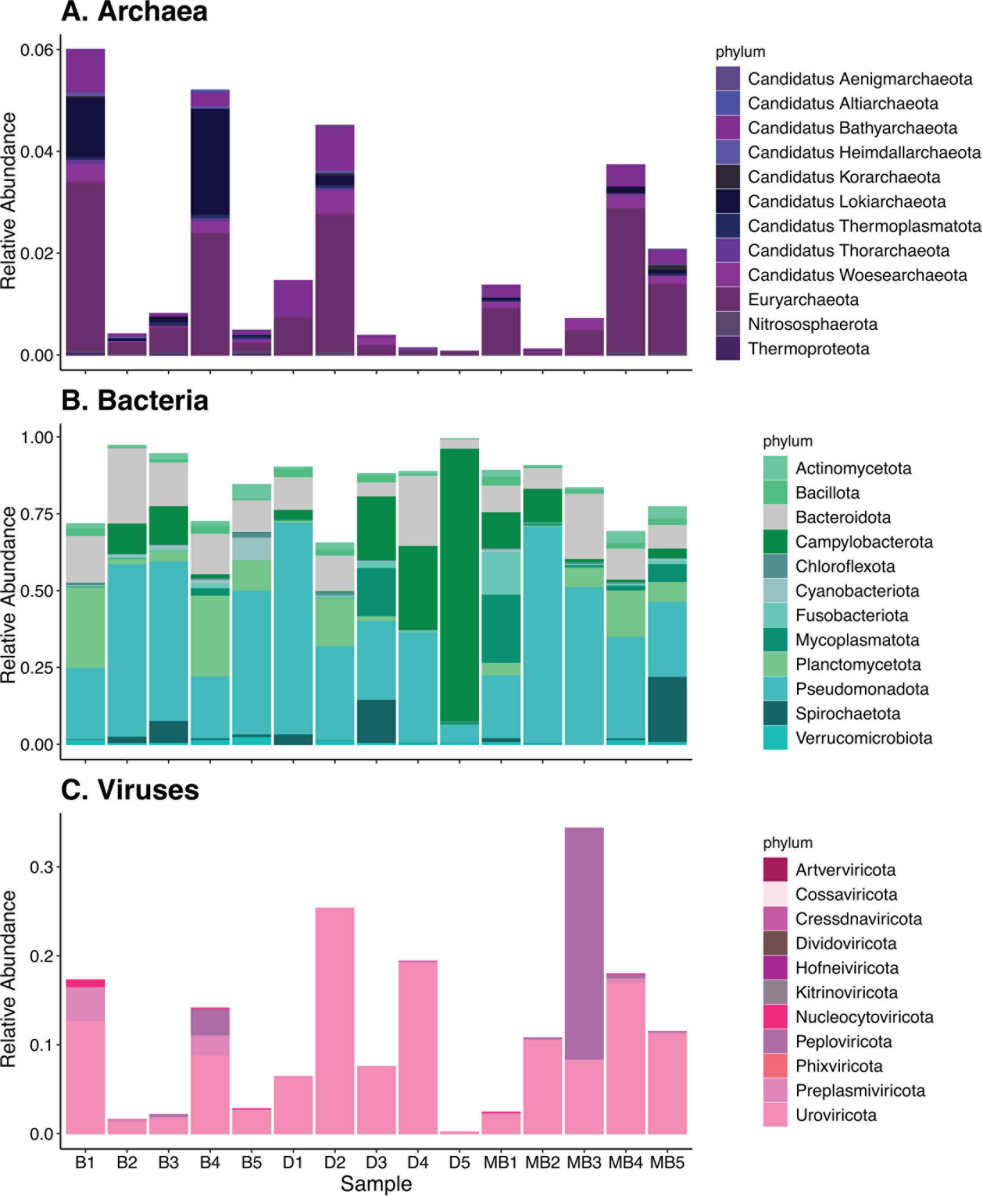
Stacked bar plots of the relative abundances of (**A**) archaeal, (**B**) bacterial, and (**C**) viral phyla comprising microbial communities associated with oyster calcifying fluid. Relative abundance was calculated at the species level within each sample.

We consistently detected archaea, bacteria, and viruses in all individual oysters (Fig. 2B). Across all individual oysters and collection locales, the highest proportion of annotated sequences were aligned to bacteria (Fig. 2B). The bacterial taxonomic diversity found in our study agrees with previous amplicon-based taxonomic assessments of calcifying fluid microbiomes. To our knowledge, this is the first study to describe archaea (Fig. 3A) and viruses (Fig. 3C) in oyster calcifying fluid. Archaea made up the smallest proportion of the microbial communities (Fig. 2B and 3A) and had phyla such as Euryarchaeota and Woesearchaeota represented in all samples (Fig. 3A). Within the bacteria, members of the phyla Pseudomonadota, Planctomycetota, and Bacteroidota were the most abundant and prevalent (Fig. 3B). Viruses, on the other hand, were relatively more abundant than archaea but less abundant than bacteria (Fig. 2B). The most abundant and prevalent phylum of viruses was Uroviricota (Fig. 3C).

**Fig 4:**
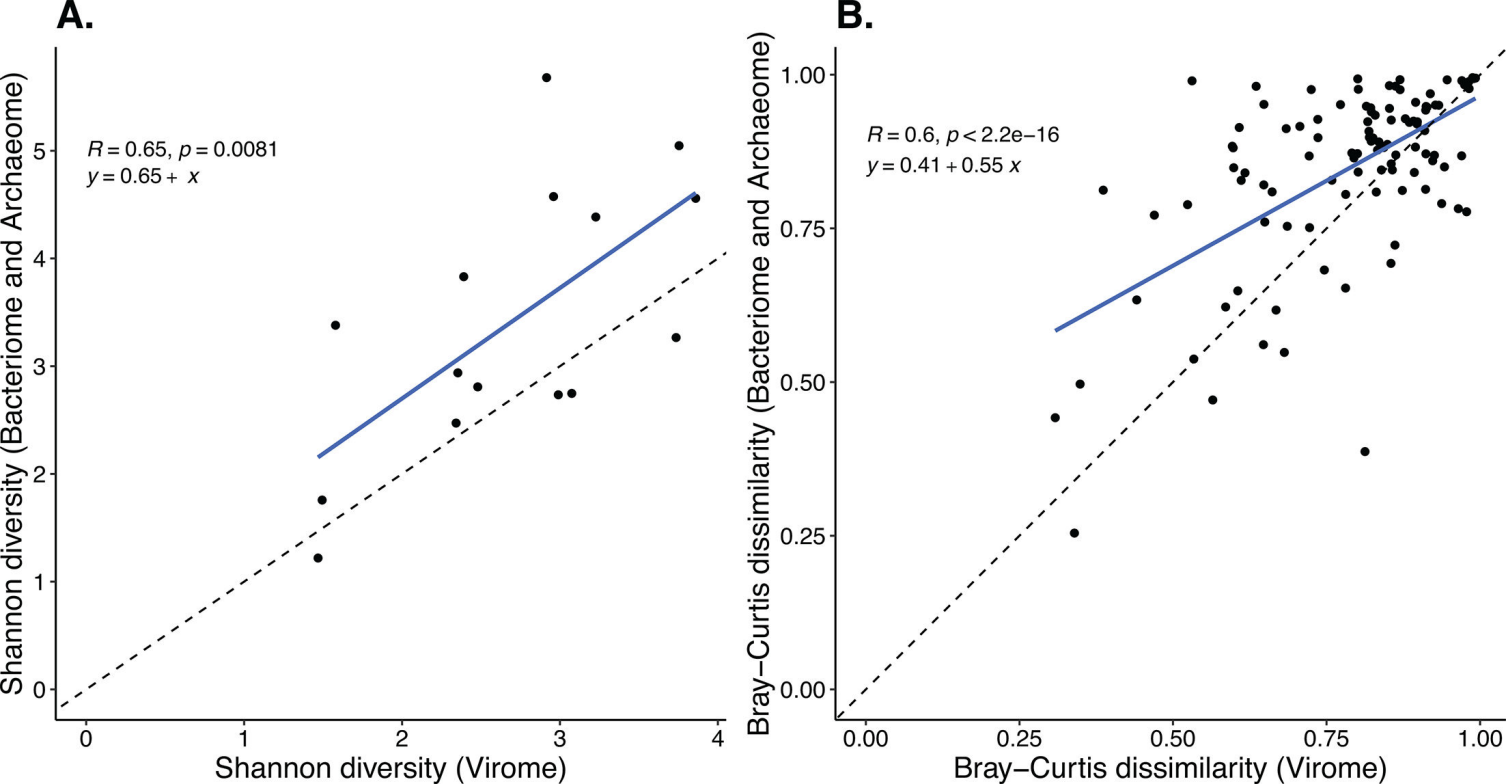
Comparison of (**A**) α-diversity (Shannon index) and (**B**) β-diversity (Bray–Curtis distance) between viral communities to combined bacterial and archaeal communities in oyster calcifying fluid. Regression lines are indicated in blue. Species-level profiles of community structures were used.

Comparison of the viral members of the community to the bacterial and archaeal members showed significant positive correlations for α-diversity (Shannon index) and β-diversity (Bray–Curtis dissimilarity) between them (*P* = 0.0081 and *P* = 2.2e^−16^, respectively; Fig. 4A and B). We found that, while α-diversity of bacterial and archaeal communities was not significantly higher than that of viral communities (Fig. S1A), β-diversity of the bacterial and archaeal communities was significantly higher than that of the viral communities (Fig. 4B; Fig. S1B). Host prediction of viral contigs revealed that the most common class of the predicted viral hosts was Gammaproteobacteria (*n* = 20) (Fig. S2). Of the phage contigs whose hosts were predicted, the majority were predicted to have a virulent (lysogenic) lifestyle (Fig. S2).

### First description of calcifying fluid microbiome functional potential using metagenomics revealed consistent profiles

**Fig 5:**
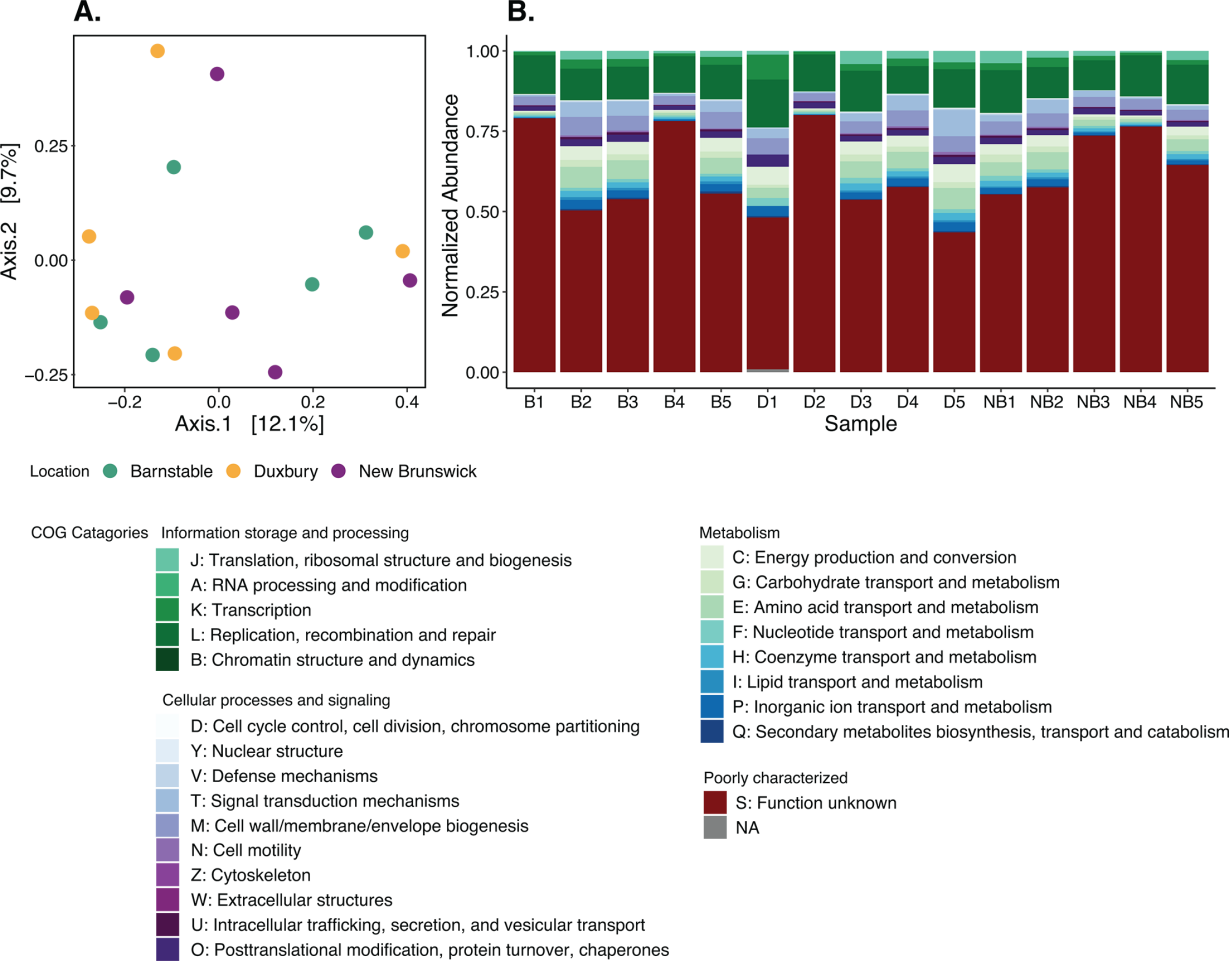
Metagenomic-predicted functional potential overview. (**A**) PCoA of Bray–Curtis dissimilarity distances of functional profiles at the seed ortholog level in calcifying fluid microbiomes colored by oyster collection location. (**B**) Stacked bar plot of the distribution of COG functional categories in oyster calcifying fluid. Gene abundances were FPKM-normalized.

Metagenomic-predicted functional potential in calcifying fluid microbiomes was not significantly different by oyster collection location (i.e., harvesting sites) (Fig. 5A; Table S3). PCoA plots of Bray–Curtis dissimilarities at the seed ortholog level showed calcifying fluid functional profiles equally dispersed across all collection locations (Fig. 5A). PERMANOVA model confirmed that calcifying fluid microbial functional profiles were not significantly different according to oyster collection location (Table S3). Clusters of Orthologous Group (COG) distributions were largely consistent across all oyster collection locations (Fig. 5B). The largest proportion of COGs in all samples was classified as having COG category S: unknown functions (Fig. 5B); the second largest proportion of COGs was classified as COG category L: replication, recombination, and repair (Fig. 5B).

### Microbiome genes associated with inorganic carbon transformations that could influence oyster calcification

**Fig 6:**
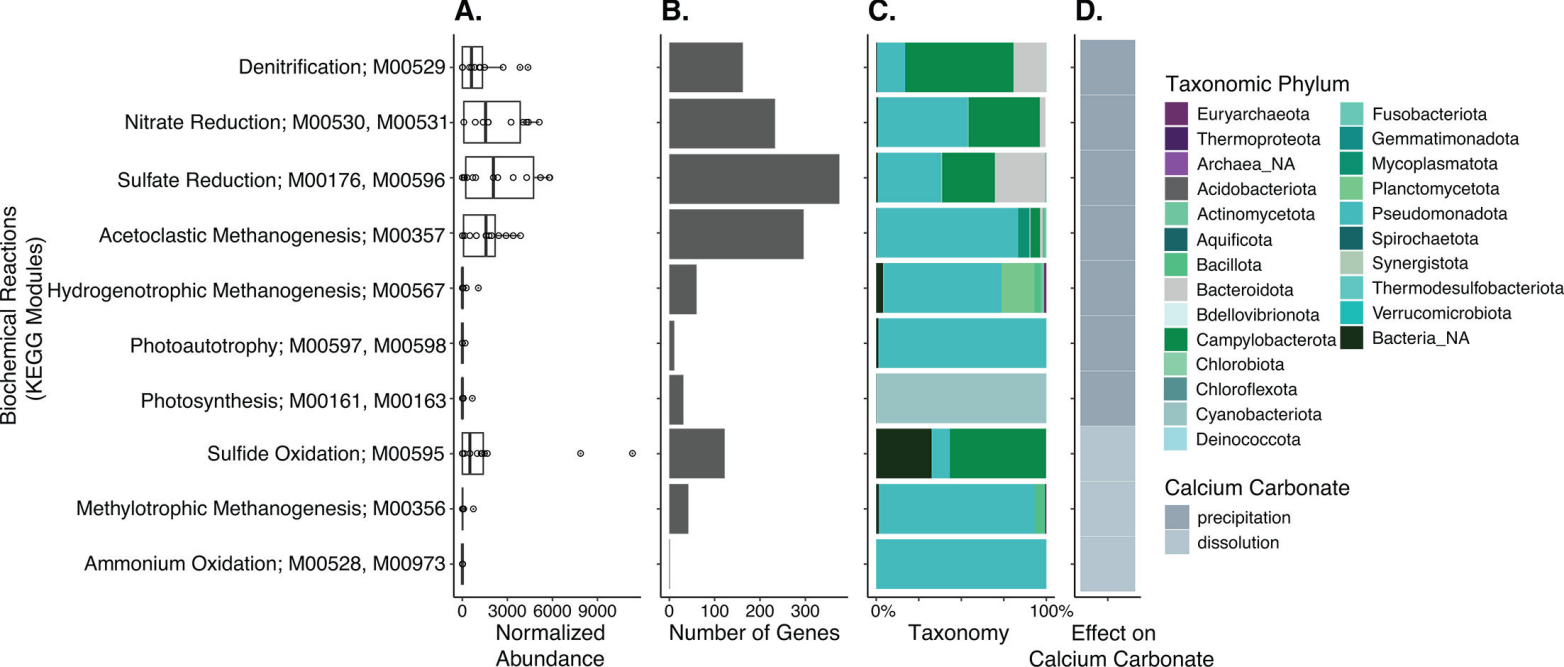
Metagenomic-predicted biochemical reactions that can play a role in the precipitation or dissolution of calcium carbonate minerals. (**A**) FPKM-normalized abundances per sample (each dot represents an individual oyster), (**B**) number of genes across all samples, (**C**) taxonomic assignments, and (**D**) putative effect of each biochemical reaction on calcium carbonate. In **A**, each dot represents the sum FPKM-normalized abundances in each sample.

To examine the whole-community functional potential to influence the carbonate system in calcifying fluid, we identified genes involved in biochemical reactions that readily alter the carbonate system in free-living microbial mats (11) using Kyoto Encyclopedia of Genes and Genomes (KEGG) modules. Out of the reactions that have previously been shown to increase [CO_3_^2-^] and Ω CaCO_3_ in marine biofilms, we found genes associated with denitrification (M00529), nitrate reduction (M00530 and M00531), sulfate reduction (M00176 and M00596), and acetoclastic methanogenesis (M00357) to have the highest representation in our metagenomes (Fig. 6A and B). On the other hand, photosynthesis (M00161 and M00163), photoautotrophy (M00597 and M00598), and hydrogenotrophic methanogenesis (M00567) were either in low abundance or absent among most samples (Fig. 6A and B). Most genes belonging to these KEGG modules were identified as Pseudomonadota, Campylobacterota, and Bacteroidota (Fig. 6C).

In contrast, we also found reactions that have previously been shown to decrease [CO_3_^2-^] and Ω CaCO_3_ in marine biofilms; the most abundant and prevalent were sulfide oxidation (M00505) (Fig. 6A and B). On the other hand, methylotrophic methanogenesis (M00356) and ammonium oxidation (M00528 and M00973) were either in low abundance or not present at all in most samples (Fig. 6A and B). Most genes belonging to these KEGG modules were identified as Pseudomonadota, Campylobacterota, and Bacteroidota (Fig. 6C).

Additionally, we found genes of two enzymes known to play key roles in bacterial calcium carbonate precipitation: carbonic anhydrase (Fig. S3A) and urease (Fig. S3B). Bacteria encode carbonic anhydrases belonging to three different genetic families: the α-, β-, and γ-classes. In our dataset, we found the α- and β-classes of bacterial carbonic anhydrases, and the β-class was the most abundant and represented across six taxonomic phyla (Fig. S3A). On the other hand, bacterial ureases are composed of either three (α-, β-, and γ-) subunits or two (α- and β-) subunits. We found all three bacterial urease subunits present in most samples (Fig. S3B).

We acknowledge that gene representation and abundance do not equal metabolic activity, but these findings suggest that there are a number of energy-conserving metabolic reactions and enzymes that, if active, could influence the pH and carbonate saturation state of the calcifying fluid and, in turn, influence net rates of calcification.

## DISCUSSION

In this study, we surveyed the taxonomic diversity and functional potential of oyster calcifying fluid microbiomes. To our knowledge, our findings present the first representation of both archaeal and viral diversity as well as functional potential using shotgun metagenomics in calcifying fluid. We found consistency in the taxonomic diversity, a number of energy-conserving metabolisms among the calcifying fluid microorganisms that could well influence calcification, as well as the presence of calcifying fluid microbial carbonic anhydrase and urease genes, key enzymes in bacterial calcium carbonate precipitation. Our findings provide a more complete picture of the taxonomy of calcifying fluid microbial communities and their functional potential to influence calcifying fluid carbon chemistry in a manner that could aid in shell formation.

### Microbiome taxonomic diversity

Previous amplicon-based studies have shown that oyster-associated microbial communities are distinct depending on oyster geographic location. For example, the microbial communities associated with oyster gills (19), hemolymph (17), and stomachs (16, 20) were significantly different among host collection sites. We expected to see a similar degree of site specificity in microbial community composition, as has been previously observed (16, 19). Our results show, however, that calcifying fluid-associated microbial communities were highly conserved across all oyster host collection sites, suggesting a higher degree of host regulation over its calcifying fluid microbiome. Calcifying fluid is in a membrane-bound, semi-permeable space with a limited exchange of ions with the surrounding seawater (21). It is reasonable to posit that the colonization of the calcifying fluid by microorganisms is more tightly governed by the oyster host in this highly regulated space.

We found archaea, bacteria, and viruses inhabiting the oyster calcifying fluid. The largest proportion of the microbiomes consisted of bacteria, and the most abundant and prevalent phyla were Pseudomonadota, Planctomycetota, and Bacteroidota. This finding is consistent with previous 16S rRNA surveys of oyster calcifying fluid that found communities dominated by the same bacterial phyla (18, 22). We also found archaea, which made up a relatively small proportion of the microbial community. Phyla such as Euryarchaeota and Woesearchaeota were present in all samples. Archaea are typically left out of oyster microbiome studies due to their low abundance (e.g., reference 23), though Pathak et al. (24), reported finding Euryarchaeota in Eastern oyster homogenate. Interestingly, members of this phylum have been found to be abundant in biofilms that precipitate carbonates (25) and could potentially contribute to calcifying fluid function. We found viruses present in calcifying fluid, the most abundant and prevalent of which were from the phylum Uroviricota. This phylum consists of a single class, Caudoviricetes, which encompasses bacterial and archaeal viruses with head-tail morphology (26). Most of the work investigating viruses associated with oysters has focused on identifying human enteric viruses (27, 28), using bacteriophages to control human enteric bacterial pathogens on oysters (29), and improving bivalve farming techniques to mitigate ostreid herpesvirus infections (30). Investigations considering viruses as integral members of oyster microbial communities are lacking. Our results build on previous studies and provide a more complete taxonomic survey of oyster calcifying fluid.

To examine patterns in microbial community composition and structure, we investigated whether there was a relationship between microbial community members that can drive diversity, that is, between viruses and their hosts (bacteria and archaea). We found that viral and bacterial profiles had significant positive correlations for both alpha and beta diversity, implying that virome and bacteriome community structures are closely related to each other in oyster calcifying fluid. This pattern has also been observed in the human gut microbiome (31–33) and is thought to be a result of viruses’ ability to affect the structure of bacterial and archaeal communities through lysis and/or integration as prophages (34). We additionally identified viral phage contigs in our dataset that were predicted to infect Gammaproteobacteria, Bacilli, and Actinomycetes among other classes of bacteria, which were also predicted to have a lysogenic lifestyle (Fig. S2). This suggests that the detected phages in our study could impact specific members of the bacterial community also detected in this study, which raises the question of how changes in viral cycles can influence the functionality of these bacterial groups in calcifying fluid. Our results highlight the need to conduct taxonomically inclusive investigations of oyster microbial communities to understand whether there are meaningful relationships between members of the microbiome that can drive diversity patterns and functional capabilities.

### Microbiome functional potential

The results presented in this study represent the first metagenomic-predicted functional potential characterization of oyster calcifying fluid. Broadly speaking, we found that overall functional potential across the whole microbial community was conserved among oyster collection sites, with COG distributions largely consistent among all individuals sampled. Our results substantially expand upon a previous study that looked at oyster calcifying fluid microbiome functional potential by using Biolog EcoPlates, assessing carbon source utilization by the community, and found that calcifying fluid microbial communities had more similar patterns of carbon utilization (i.e., functional potential) across oyster populations than gut communities (35). Many genes were poorly characterized and of “unknown function” (likely a result of the bias in public repositories toward model organisms, vertebrates, animals used in medical research, and livestock [36]). Samples with a higher proportion of unknown functions (e.g., B1, B4, D2, NB3, and NB4) also had relatively higher viral loads (Fig. 3C). This is consistent with previous findings which show that marine metagenomes with the largest ratio of genes with unknown functions are those enriched with viral sequences (37). The second most abundant COG category was L: “replication, recombination and repair,” which is usually considered as marker of mobile genetic elements and has been associated with genetic plasticity of microbial communities (38). Genetic plasticity in a microbial community can result from living under constant selection by the host immune system (39). Additionally, members of the calcifying fluid microbiome are potentially acquired from the environment, and having free-living and host-associated life stages exposes microorganisms to variable environments which can result in a high degree of genetic plasticity (38).

### Microbiome genes associated with inorganic carbon transformations

Among microbial metabolic reactions that result in increased [CO_3_^2-^] and Ω CaCO_3_ in favor of calcification, we found genes associated with sulfate reduction (M00176 and M00596), denitrification (M00529), and nitrate reduction (M00530 and M00531). This finding further supports the hypothesis that sulfate-reducing bacteria may be involved in the formation of chalky deposits in oysters (6, 7), and it agrees with a previous study that found predicted genes involved in sulfate reduction and dissimilatory nitrate reduction in oyster calcifying fluid using functional predictions from marker genes (18). We also found genes involved in acetoclastic (M00357) and hydrogenotrophic (M00567) methanogenesis. Though these anaerobic reactions have been previously shown to aid in microbial-mediated calcification (11), they have not been previously identified in oyster calcifying fluid. Genes related to photosynthesis (M00161 and M00163) and photoautotrophy (M00597 and M00598) were either in low abundance or absent in most samples. Considering calcifying fluid is located under the shell, where sunlight does not penetrate, it is not surprising these sunlight-dependent reactions are not present in our dataset.

We also considered microbial metabolic reactions that result in decreased [CO_3_^2-^] and Ω CaCO_3_ in favor of decalcification. We found genes associated with sulfide oxidation (M00505) present in most samples, while methylotrophic methanogenesis (M00356) and ammonium oxidation (M00528 and M00973) were either in low abundance or not present at all in most samples. Genes associated with these biochemical reactions have not been previously identified in oyster calcifying fluid. Even though calcifying fluid is known for its role in the calcification of shell, it also acts as a buffering system to neutralize products of anaerobic metabolism, which accumulate in periods of shell closure, by the dissolution of previously deposited shell (2). Therefore, it is important to consider microbial metabolic reactions that could aid in decalcification as well as calcification.

We also found genes coding for bacterial carbonic anhydrase (EC 3.5.1.5) and urease (EC 4.2.1.1), two key enzymes in microbially mediated calcification. Dhami et al. (40) showed that carbonic anhydrase, a zinc-containing enzyme that catalyzes the reversible hydration of carbon dioxide: CO_2_ + H_2_O ↔ HCO_3_^−^ + H^+^, plays a role in increasing bicarbonate concentration, while urease, a nickel-containing metalloenzyme that catalyzes the hydrolysis of urea: (NH_2_)_2_CO + H_2_O → CO_2_ + 2NH_3_, aids in maintaining the alkaline pH that promotes calcium carbonate precipitation. The synergistic activity of carbonic anhydrase and urease optimized the rate of calcification in bacterial cultures (40, 41). It is possible for these two enzymes to play a similar role in oyster calcifying fluid. The functional role and synergistic activity of bacterial carbonic anhydrase and urease in calcifying fluid should also be further investigated due to their prevalence among oyster populations in our study, and thus potential importance in oyster calcification.

### Conclusions

Our investigation into the taxonomic diversity and functional potential of oyster calcifying fluid microbiomes through shotgun metagenomics provides a deeper understanding of both archaeal and viral diversity within the calcifying fluid than previously documented. Notably, the remarkable consistency observed in taxonomic diversity underscores the stability of these microbial communities among oysters collected across three different sites, two of which are in Massachusetts and the third in the Canadian province of New Brunswick. This is the first shotgun metagenomic representation of the functional potential of the calcifying fluid microbiome, and the results underscore the very likely possibility that microbial activity within the calcifying fluid can support or hinder oyster calcification. The identification of various energy-conserving metabolisms that can alter the carbonate system hints at the potential influence of the microbiome on host calcification. The representation of microbial carbonic anhydrase and urease genes further emphasizes the functional significance of these microbiomes in mediating inorganic carbon chemistry, crucial for shell formation. However, these are not measures of metabolic activity, and future studies should endeavor to test whether these metabolic processes are occurring within the calcifying fluid and the extent to which they support or hinder net calcification.

## MATERIALS AND METHODS

### Oyster collection

We purchased adult Eastern oysters (88- to 100-mm shell length), *C. virginica*, from three different oyster farms within the span of 1 week the summer of 2022: Thatch Island Oysters (41°42′37.6″N 70°18′18.5″W) in Barnstable, Massachusetts, USA (*n* = 5), Island Creek Oysters (42°02′10.7″N 70°40′12.4″W) in Duxbury, Massachusetts, USA (*n* = 5), and Chebooktook Oysters (46°28′59.6″N 64°39′00.0″W) in New Brunswick, Canada (*n* = 5). The three sites are part of distinct current systems, and the two oyster farms in the USA are in the Gulf of Maine, while the Canadian oyster farm is in the Gulf of St. Lawrence; the Gulf of Maine and the Gulf of St. Lawrence experience different temperature, salinity, and nutrient regimes (42, 43). At a more local scale, the sites have distinct geological and sedimentation patterns as well as freshwater input sources (44–46).

The oysters were transported on ice from their purchase locations to Biological Laboratories at Harvard University, where they were placed in a rearing tank with filtered seawater and fed 20 mL of a 1% Shellfish Diet 1800 solution, following best practices outlined in Helm Bourne, (47). After 24 hours of recovery, calcifying fluid was collected (method described below). Transportation times varied between 3 and 8 hours. The short transport times, cold conditions, and immersion recovery with feed minimized stress and resulted in 0% mortality rate.

The seawater in our rearing tank was collected from Hampton State Pier in Hampton, New Hampshire, and had a salinity of 34 PSU (practical salinity unit). Seawater was UV-sterilized, filtered through a 30-µm filter, chilled to 6°C, and stored in 7,500-L polyurethane tank onsite. The seawater was allowed to warm up from 6°C to 18°C before putting the oyster in the rearing tank.

### Calcifying fluid sampling

Since calcifying fluid is sealed inside the oysters, it was highly unlikely for contamination to occur during transport. To access the calcifying fluid, we developed a method to penetrate the outer shell without puncturing the mantle. First, we used a DREMEL tool (model 8220; Dremel, Racine, WI, USA) with a model 225 flexible shaft extension to drill 2-mm diameter holes on the left valve, about 2 cm away from the hinge, of each oyster. Prior to drilling, we used 70% ethanol and KimWipes to sterilize the tool bits and the shell surface and to remove excess calcium carbonate powder from the drill site. To ensure the mantle tissue was not damaged, we visually inspected the mantle through the drilled hole. To collect calcifying fluid, a sterile 5-mL syringe and a gavage needle (a sterile, flexible, plastic tubing needle with a rounded-silicon tip) were used to prevent puncturing the mantle. Approximately 0.5 mL of calcifying fluid was collected from 15 oysters. Each sample was put it into a 2-mL cryovial, flash-frozen in liquid nitrogen, and stored at −80°C until DNA extraction.

### Nucleic acid preparation and sequencing

DNA was extracted from calcifying fluid samples using the DNeasy PowerLyzer PowerSoil Kit (Qiagen, Valencia, CA, USA) following the manufacturer’s protocol, with the addition of three ethanol washes to remove excess salts prior to solubilization of DNA in nuclease-free water. Sequencing libraries were prepared using the Illumina DNA Prep Library Preparation Kit and sequenced on the Illumina NovaSeq S4 System (paired end, 150 bp) at the Bauer Core Facility at Harvard University. To overcome the high ratios of host DNA relative to microbial DNA, our approach was to sequence deeply and bioinformatically remove host reads (see below for details).

### Shotgun metagenomic sequence analysis

Shotgun metagenomic sequencing allows for the assessment of all microbial domains (Bacteria, Eukarya, Archaea) and viruses for a complete community analysis (48, 49)(50). Paired-end reads were quality-filtered using Trim Galore v.0.6.10 (51) to trim, remove adapter content, and to ensure a minimum PHRED score of 30 and were at least 120-bp long. The quality of filtered reads were assessed with FastQC v.0.12.1 (52). Oyster host reads were removed by using Bowtie2 v2.3.2 in very sensitive local mode (53) to align quality-filtered reads to the *C. virginica* and *Crassostrea gigas* genomes and then filtered out with SAMtools v.1.17 (54). Non-oyster reads were used to assemble metagenomic contigs, independently for each sample, with metaSPAdes v.3.15.5 (55). The assembled contigs were then aligned to the *C. virginica* genome using BLAST to detect oyster contigs, which were filtered out using a Python script. The remaining (1,197,746) non-oyster contigs were used for downstream taxonomic and functional potential analyses.

### Coverage assessment

Redundancy estimation of non-oyster reads was calculated using Nonpareil (56) v.3.304 with parameters -T kmer -X 1000000. Nonpareil curves were built using the R package Nonpareil in RStudio, R v4.3.1. Rarefaction curves of the number of species versus the number of sequences in each oyster collection location were calculated using the Vegan v2.6-4 (57) in RStudio, R v4.3.1. Taxonomic annotation was conducted at the contig level for the rarefaction analysis.

### Metagenomic-predicted taxonomy

Non-oyster contigs were assigned taxonomic labels using MMseqs2 taxonomy v.14.7 (58) against the Uniref90 database. MMseqs2 taxonomy extracts all possible proteins from each contig, assigns taxonomic labels to each protein, and then determines taxonomic identity of the contig in question by weighed voting (58). Contigs identified as Eukaryotic were filtered out, to ensure no oyster contamination remained. CoverM v. 0.6.1 (https://github.com/wwood/CoverM) (parameters: –min-read-aligned-percent 75% –min-read-percent-identity 95%) was used to determine the relative abundance (coverage) of each contig within in each metagenomic sample. Relative abundances of contigs were calculated as the percentage of total sample reads mapping to each contig.

### Metagenomic-predicted functional potential

Sequencing depth precluded the binning and study of MAGs in our study but should remain the focus of future inquiries. We focused our efforts in investigating the contigs that were successfully assembled. Non-oyster contigs were used to predict open reading frames (ORFs) using Prodigal v.2.6.3 (59) and annotated using DIAMOND v.2.1.8 (60) in eggNOG-mapper v.2.1.11 (61). EggNOG-mapper uses orthologous groups and phylogenies from the eggNOG database to transfer functional information from fine-grained orthologs to the identified ORFs. The functional annotations per query provided by eggNOG-mapper include predicted protein name; KEGG pathways, modules, and orthologs (62); Gene Ontology labels (63); EC numbers, BiGG reactions (64); CAZy terms (65); COG functional categories (66); eggNOG OGs; and text descriptions at all taxonomic levels. Quality-filtered non-oyster reads were then mapped to the ORFs using Bowtie2 v2.3.2 in very sensitive local mode (53) to estimate abundance. Read counts of ORFs were normalized by gene length and sample sequencing depth as fragments per kilobase million (FPKM).

### Statistical analysis

To compare taxonomic diversity among oyster collection locations, a Bray–Curtis dissimilarity matrix was computed at the species level, using the distance function in Phyloseq (67), and used to run a PERMANOVA, with location as factor, with 999 permutations using the adonis2 function in v2.6-4 (57). Homogeneity of group dispersions was tested using betadisper function in Vegan v2.6-4 (57). To visualize the beta diversity of the different oyster locations, we used a PCoA plot using the plot_ordination function in Phyloseq (67) and ggplot2 (68). We visualized taxonomic distribution across samples with stacked bar plots using ggplot2 (68).

To investigate the relationship between taxonomic domains in the community, we looked at the diversity of archaea and bacteria (grouped together) and viruses. We used α-diversity (Shannon diversity) and β-diversity (Bray–Curtis distance) metrics. Shannon diversity at the species level was calculated for each sample using the diversity function in Vegan v2.6-4 (57) and then plotted as a scatter plot. Bray–Curtis distance at the species level was computed using the vegdist function in Vegan v2.6-4 (57), and pairwise comparisons were then plotted as scatter plot. We computed linear regression models to assess whether there was a significant relationship between the viral vs bacterial and archaeal members of the community. To further explore this association, we examined contigs identified as phages using PhaBox (69) to identify the lifestyle (lytic vs temperate), their potential Prokaryotic host, and taxonomic assignment. Contigs 3-kB long or greater were used in this portion of the analysis (*n* = 81). Results were visualized using ggplot2 (68) in R studio.

To compare functional potential among the three collection locations, a Bray–Curtis dissimilarity matrix was computed at the seed ortholog level, using the distance function in Phyloseq (67), and used to run a PERMANOVA, with location as factor, with 999 permutations using the adonis2 function in Vegan v2.6–4 (57). Homogeneity of group dispersions was tested using betadisper function in Vegan v2.6–4 (57). To visualize protein dissimilarities between the different oyster locations, we used a PCoA plot using the plot_ordination function in Phyloseq (67) and ggplot2 (68).We also visualized the distribution of COG annotations with a stacked bar plot in ggplot2 (68).

To examine the whole-community functional potential to influence the carbonate system in calcifying fluid, first, we identified genes involved in biochemical reactions that readily alter the carbonate system in free-living microbial mats (11) using KEGG modules. KEGG modules are functional units representing gene sets of functional relevance. For example, to assess if there were genes related to photosynthesis in our dataset, we searched for genes assigned to KEGG modules involved in photosynthesis like modules M00161 and M00163, which encompass genes in photosystems II and I, respectively. Common microbial-mediated reactions that can increase [CO_3_^2-^] and Ω CaCO_3_ include denitrification (KEGG module number: M00529), nitrate reduction (M00530 and M00531), sulfate reduction (M00176 and M00596), acetoclastic methanogenesis (M00357), hydrogenotrophic methanogenesis (M00567), photosynthesis (M00161 and M00163), and photoautotrophy (M00597 and M00598). We also identified genes involved in microbial-mediated reactions that decrease [CO_3_^2-^] and Ω CaCO_3_ including sulfide oxidation (KEGG module number: M00595), methylotrophic methanogenesis (M00356), and ammonium oxidation (M00528 and M00973). We visualized the normalized abundance of genes associated with the KEGG module numbers and number of genes in each sample as well as their assigned taxonomy in ggplot2 (68) in R.

Second, we identified enzymes that are known to promote microbially induced calcium carbonate precipitation. Genes coding for carbonic anhydrase and urease were identified using KEGG EC numbers 3.5.1.5 and 4.2.1.1, respectively. We visualized the different classes of each of these enzymes and their taxonomic assignment using a box and whisker plot in ggplot2 (68).

## Data Availability

Metagenomic reads produced in this study are available under NCBI BioProject accession number PRJNA1108182.

## SUPPLEMENTAL MATERIAL

Supplemental material aem.01094-24-s0001.pdf Tables S1 to S4; Figures S1 to S3.

ASM does not own the copyrights to Supplemental Material that may be linked to, or accessed through, an article. The authors have granted ASM a non-exclusive, world-wide license to publish the Supplemental Material files. Please contact the corresponding author directly for reuse.
