# Supplementary material for: Taxonomic diversity and functional potential of microbial communities in oyster calcifying fluid
Source: Appl Environ Microbiol. 2024 Dec 12;91(1):e01094-24. doi: 10.1128/aem.01094-24 (PMC11784444; doi:10.1128/aem.01094-24)
Supplement: Supplemental material — Tables S1 to S4; Figures S1 to S3. [file aem.01094-24-s0001.pdf]

Supplemental information for: Taxonomic diversity and functional potential of microbial communities in oyster calcifying fluid

Andrea Unzueta-Matínez and Peter R. Girguis

Table S1. Sample metadata. All samples used in this study and the metadata (oyster farm geographical origin, oyster species, tissue sampled, library size, NCBI SRA accession numbers for reads, and assembly methods) are shown.

Sample Name	Oyster farm of origin	Geographical origin	Latitude and longitude	Host species	Tissue type sampled	NCBI SRA Accession	NCBI BioProject	Total read pairs	Assembly method and version
mollusk_microbiome_metagenome_Duxbury_replicate1	Island Creek Oysters	USA: Duxbury	42.036306 N, 70.670111 W	*Crassostrea virginica*	calcifying fluid	SAMN41228059	PRJNA1108182	128057173	metaSPAdes v3.15.5
mollusk_microbiome_metagenome_Duxbury_replicate2	Island Creek Oysters	USA: Duxbury	42.036306 N, 70.670111 W	*Crassostrea virginica*	calcifying fluid	SAMN41228060	PRJNA1108182	212235964	metaSPAdes v3.15.5
mollusk_microbiome_metagenome_Duxbury_replicate3	Island Creek Oysters	USA: Duxbury	42.036306 N, 70.670111 W	*Crassostrea virginica*	calcifying fluid	SAMN41228061	PRJNA1108182	225549944	metaSPAdes v3.15.5
mollusk_microbiome_metagenome_Duxbury_replicate4	Island Creek Oysters	USA: Duxbury	42.036306 N, 70.670111 W	*Crassostrea virginica*	calcifying fluid	SAMN41228062	PRJNA1108182	141165979	metaSPAdes v3.15.5
mollusk_microbiome_metagenome_Duxbury_replicate5	Island Creek Oysters	USA: Duxbury	42.036306 N, 70.670111 W	*Crassostrea virginica*	calcifying fluid	SAMN41228063	PRJNA1108182	203091351	metaSPAdes v3.15.5
mollusk_microbiome_metagenome_Barnstable_replicate1	Thatch Island Oysters	USA: Barnstable	41.710444 N, 70.305139 W	*Crassostrea virginica*	calcifying fluid	SAMN41228064	PRJNA1108182	233198766	metaSPAdes v3.15.5
mollusk_microbiome_metagenome_Barnstable_replicate2	Thatch Island Oysters	USA: Barnstable	41.710444 N, 70.305139 W	*Crassostrea virginica*	calcifying fluid	SAMN41228065	PRJNA1108182	187133955	metaSPAdes v3.15.5
mollusk_microbiome_metagenome_Barnstable_replicate3	Thatch Island Oysters	USA: Barnstable	41.710444 N, 70.305139 W	*Crassostrea virginica*	calcifying fluid	SAMN41228066	PRJNA1108182	49100072	metaSPAdes v3.15.5
mollusk_microbiome_metagenome_Barnstable_replicate4	Thatch Island Oysters	USA: Barnstable	41.710444 N, 70.305139 W	*Crassostrea virginica*	calcifying fluid	SAMN41228067	PRJNA1108182	124751654	metaSPAdes v3.15.5
mollusk_microbiome_metagenome_Barnstable_replicate5	Thatch Island Oysters	USA: Barnstable	41.710444 N, 70.305139 W	*Crassostrea virginica*	calcifying fluid	SAMN41228068	PRJNA1108182	97206388	metaSPAdes v3.15.5
mollusk_microbiome_metagenome_NewBrunswick_replicate1	Chebooktook Oysters	Canada: New Brunswick	46.483222 N, 64.650000 W	*Crassostrea virginica*	calcifying fluid	SAMN41228069	PRJNA1108182	179955638	metaSPAdes v3.15.5
mollusk_microbiome_metagenome_NewBrunswick_replicate2	Chebooktook Oysters	Canada: New Brunswick	46.483222 N, 64.650000 W	*Crassostrea virginica*	calcifying fluid	SAMN41228070	PRJNA1108182	78122548	metaSPAdes v3.15.5
mollusk_microbiome_metagenome_NewBrunswick_replicate3	Chebooktook Oysters	Canada: New Brunswick	46.483222 N, 64.650000 W	*Crassostrea virginica*	calcifying fluid	SAMN41228071	PRJNA1108182	86277511	metaSPAdes v3.15.5
mollusk_microbiome_metagenome_NewBrunswick_replicate4	Chebooktook Oysters	Canada: New Brunswick	46.483222 N, 64.650000 W	*Crassostrea virginica*	calcifying fluid	SAMN41228072	PRJNA1108182	116741116	metaSPAdes v3.15.5
mollusk_microbiome_metagenome_NewBrunswick_replicate5	Chebooktook Oysters	Canada: New Brunswick	46.483222 N, 64.650000 W	*Crassostrea virginica*	calcifying fluid	SAMN41228073	PRJNA1108182	125089193	metaSPAdes v3.15.5

Table S2. Statistical analysis of the effect of location on species-level Bray-Curtis dissimilarities of oyster calcifying fluid microbial communities. (A) Results of a permutational test for homogeneity of group dispersions (betadisper). (B) Results of PERMANOVA (Adonis), using 999 permutations.

A. Homogeneity of group dispersions test:

Factor	DF	Sum of Squares	Mean Squares	F	Pr(>F)	significance
Location	2	0.011853	0.0059266	0.6883	0.5212	NS
Residuals	12	0.103319	0.0086099			

B. PERMANOVA:

Factor	Df	Sum of Squares	F	R2	Pr(>F)	significance
Location	2	0.7757	1.0851	0.15315	0.301	NS
Residual	12	4.2892		0.84685		
Total	14	5.0649		1		

Table S3. Statistical analysis of the effects of location on gene profile Bray-Curtis dissimilarities of oyster calcifying fluid functional potential. (A) Results of a permutational test for homogeneity of group dispersions (betadisper). (B) Results of PERMANOVA (Adonis), using 999 permutations.

A. Homogeneity of group dispersions test:

Factor	DF	Sum of Squares	Mean Squares	F	Pr(>F)	significance
Location	2	0.0023334	0.0011667	0.6394	0.5447	NS
Residuals	12	0.0218967	0.0018247			

B. PERMANOVA:

Factor	Df	Sum of Squares	F	R2	Pr(>F)	significance
Location	2	0.9366	0.14615	1.027	0.337	NS
Residual	12	5.4717	0.85385			
Total	14	6.4083	1			

Table S4. Overview of microbial reactions that can change local environment to support or hinder calcification.

Microbial metabolic process	Reaction	Supports (+) or hinders (-) calcification	KEGG module number
denitrification	$NO_3^- \rightarrow NO_2^- \rightarrow NO \rightarrow N_2O \rightarrow N_2$	+	M00529
nitrate reduction	$NO_3^- \rightarrow NO_2^- \rightarrow NH_4^+$	+	M00530, M00531
sulphate reduction	$SO_4^{2-} \rightarrow APS \rightarrow SO_3^{2-} \rightarrow H_2S$	+	M00176, M00596
acetoclastic methanogenesis	$C_2H_3O_2^- \rightarrow CH_4$	+	M00357
hydrogenotrophic methanogenesis	$CO_2 \rightarrow CH4$	+	M00567
photosynthesis	$CO_2 \rightarrow C_6H_{12}O_6$	+	M00161, M00163
photoautotrophy	$CO_2 \rightarrow CH_2O$	+	M00597, M00598
sulfide oxidation	$S_2O_3^{2-} \rightarrow SO_4^{2-}$	-	M00595
methylotrophic methanogenesis	$CH_3OH \rightarrow CH_4$	-	M00356
ammonium oxidation	$NH_4 \rightarrow NO_2^-$	-	M00528, M00973

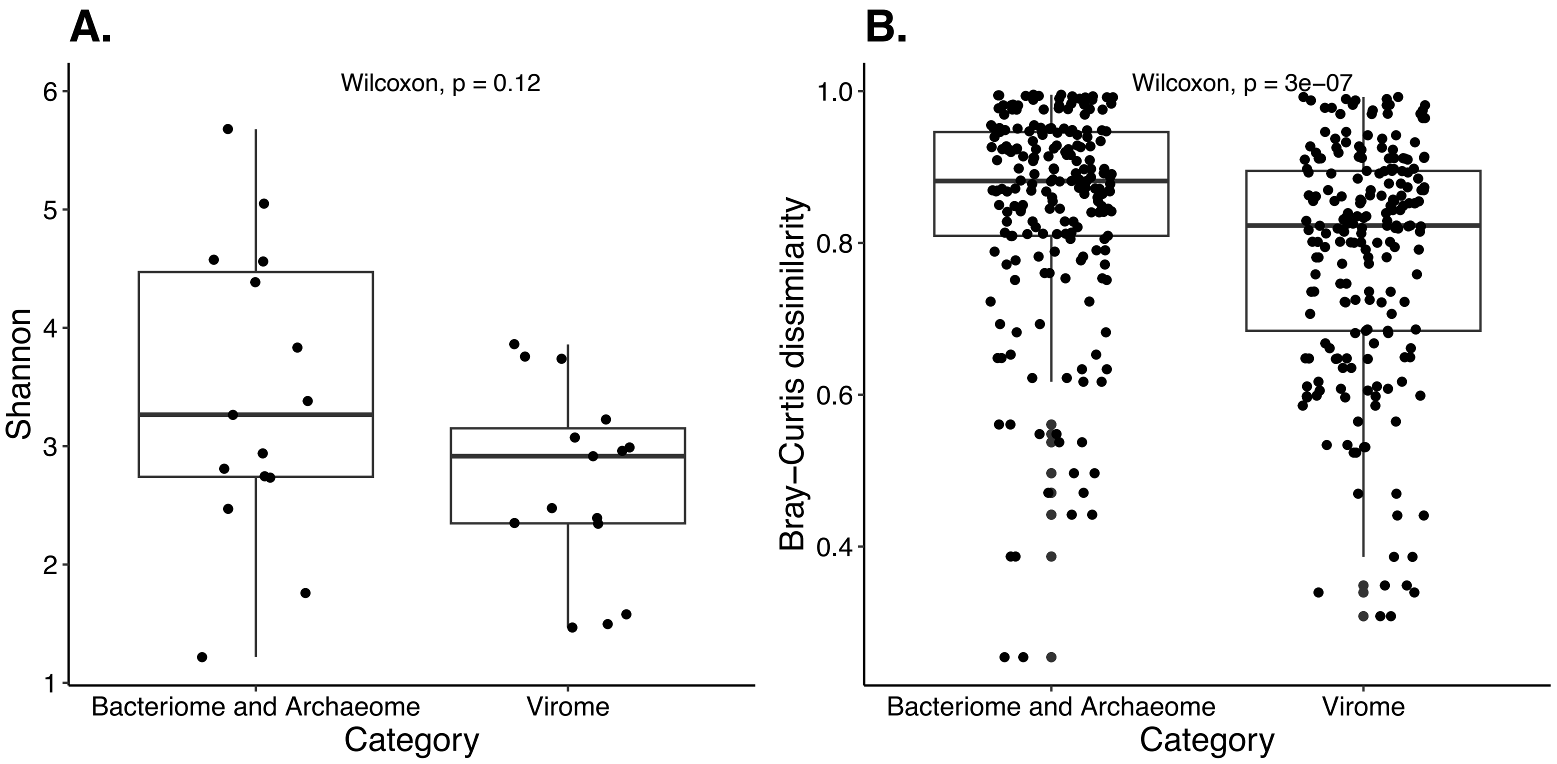

Figure S1. Boxplot comparing calcifying fluid Viral communities to Bacterial and Archaeal communities with (A) α-diversity (Shannon index) and (B) β-diversity (Bray-Curtis dissimilarity). P-value was obtained by the Wilcoxon rank-sum test (two-sided).

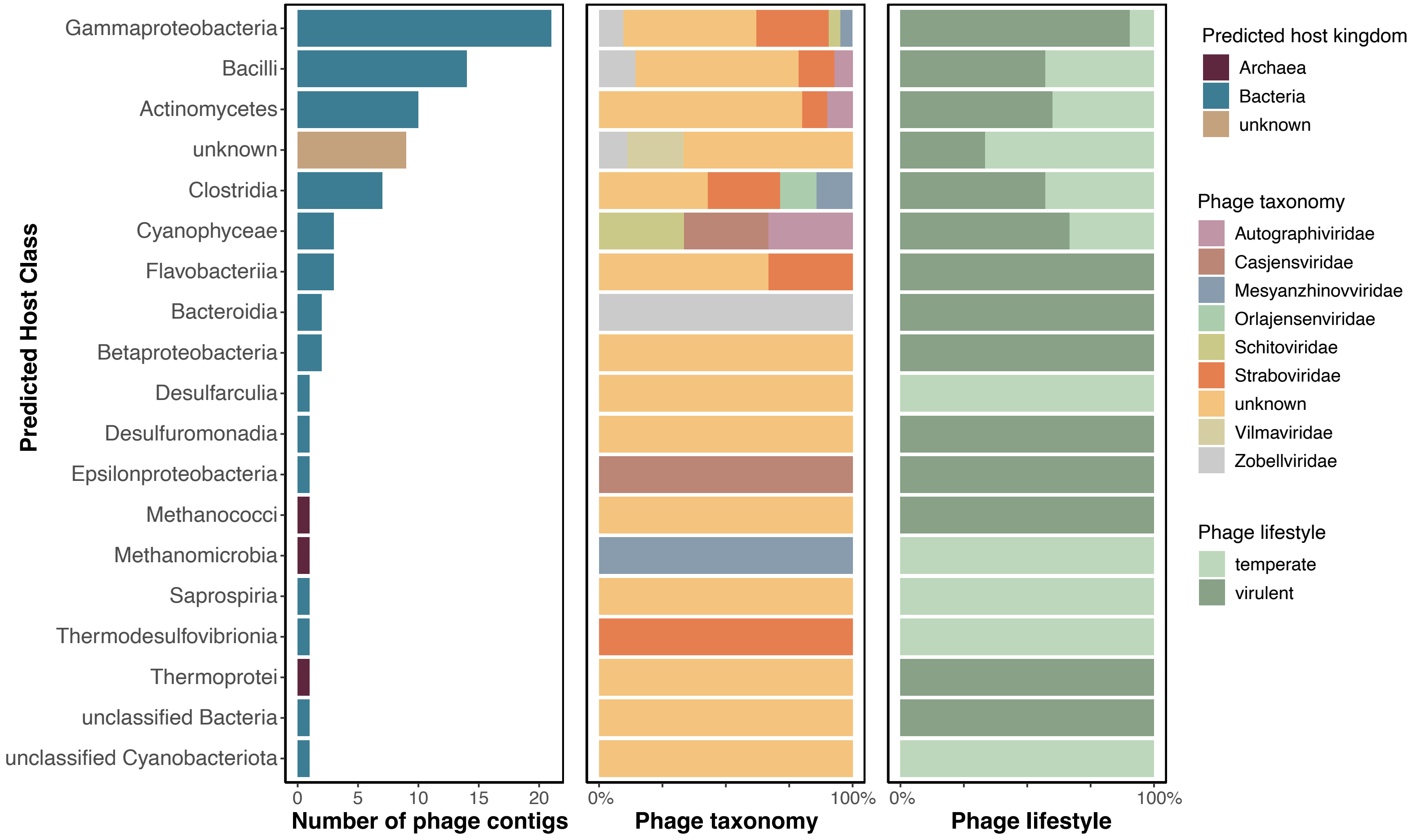

Figure S2. Number of phage contigs, phage taxonomy, and the ratio of virulent and temperate phages for each predicted host (at the class level).

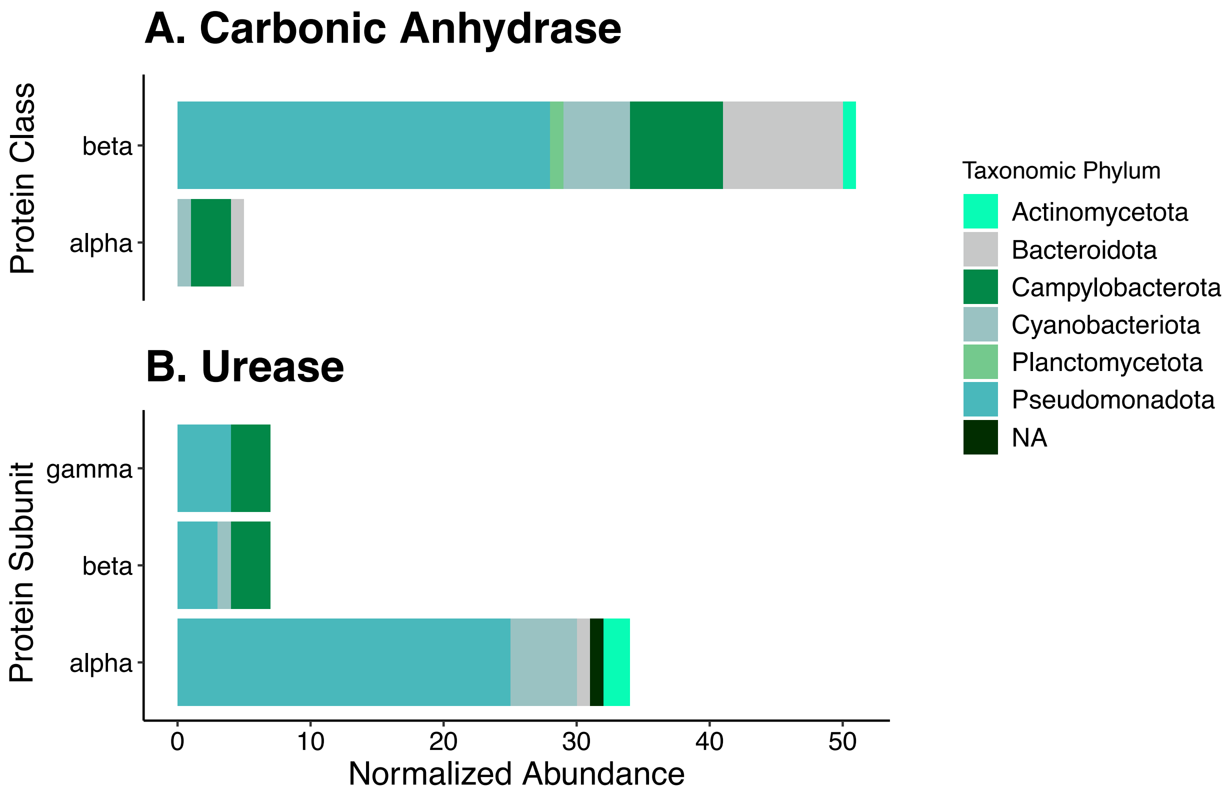

Figure S3. Key enzymes, (A) carbonic anhydrase and (B) urease, in bacterial $CaCO_3$ precipitation identified in metagenomic-predicted functional potential of calcifying fluid microbiomes. Abundances are FPKM normalized.
